# Supplementary material for: Midkine Is a Potential Therapeutic Target of Tumorigenesis, Angiogenesis, and Metastasis in Non-Small Cell Lung Cancer
Source: Cancers (Basel). 2020 Aug 24;12(9):2402. doi: 10.3390/cancers12092402 (PMC7563676; doi:10.3390/cancers12092402)
Supplement: Supplementary file 1 [file cancers-12-02402-s001.zip › Figure S1-S15.pdf]

Supplementary Materials: Midkine is a Potential Therapeutic Target of Tumorigenesis, Angiogenesis, and Metastasis in Non-Small Cell Lung Cancer

Dong Hoon Shin, Jeong Yeon Jo, Sun Ha Kim, Minyoung Choi, Chungyong Han, Beom K. Choi and Sang Soo Kim

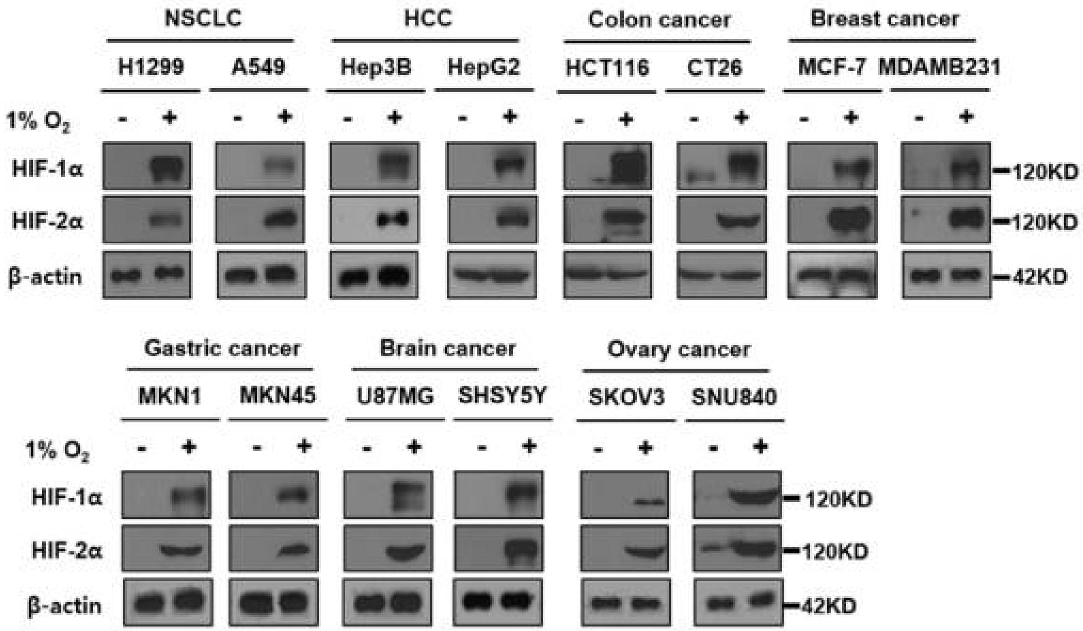

Figure S1. Expression levels HIF-1α and HIF-2α. H1299, A549 (NSCLC); Hep3B, HepG2 (HCC); HCT116, CT26 (Colon cancer); MCF7, MDAMB231 (Breast cancer); MKN1, MKN45 (Gastric cancer); U87MG, SHSY5Y (Brain cancer); and SKOV3, SNU840 (Ovary cancer) cells were incubated for 16 h under normoxic (20% O_2) or hypoxic (1% O_2) conditions. HIF-1α, HIF-2α and β-actin expressions were analyzed by immunoblotting.

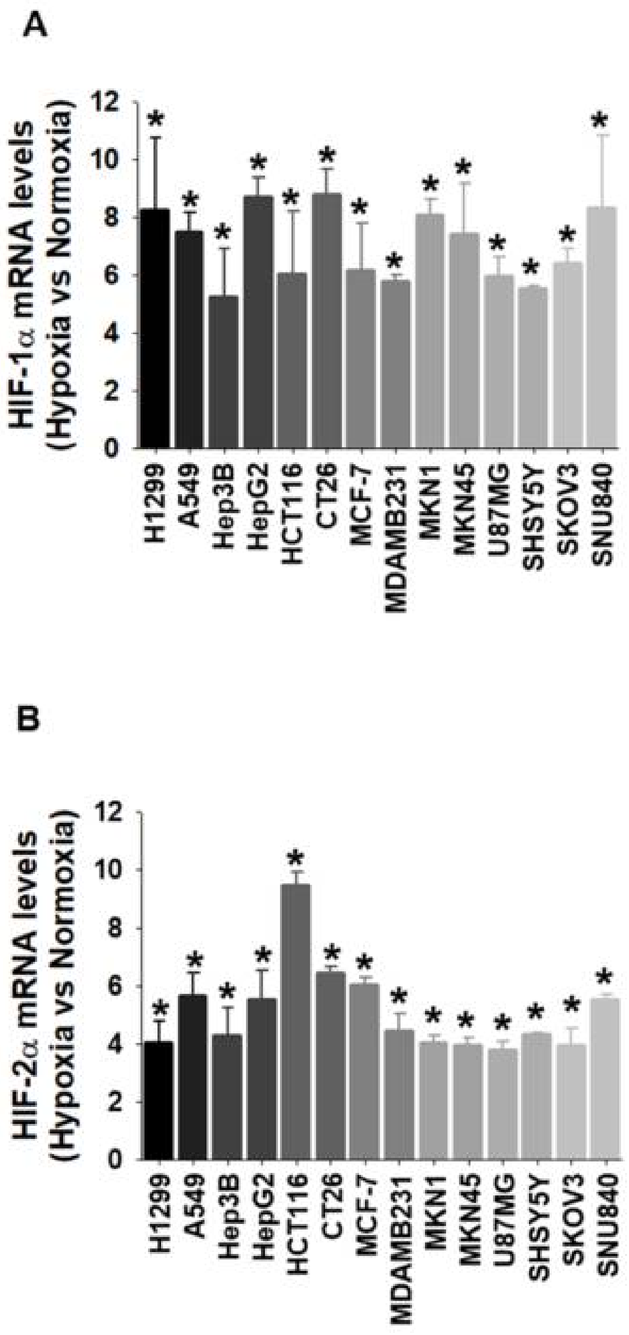

Figure S2. Measurements of mRNA levels of HIF-1α and HIF-2α under the same conditions as mentioned in Figure S1. Student's *t*-test was performed and values displayed as average ±SEM; $n = 6$, * $p < 0.05$.

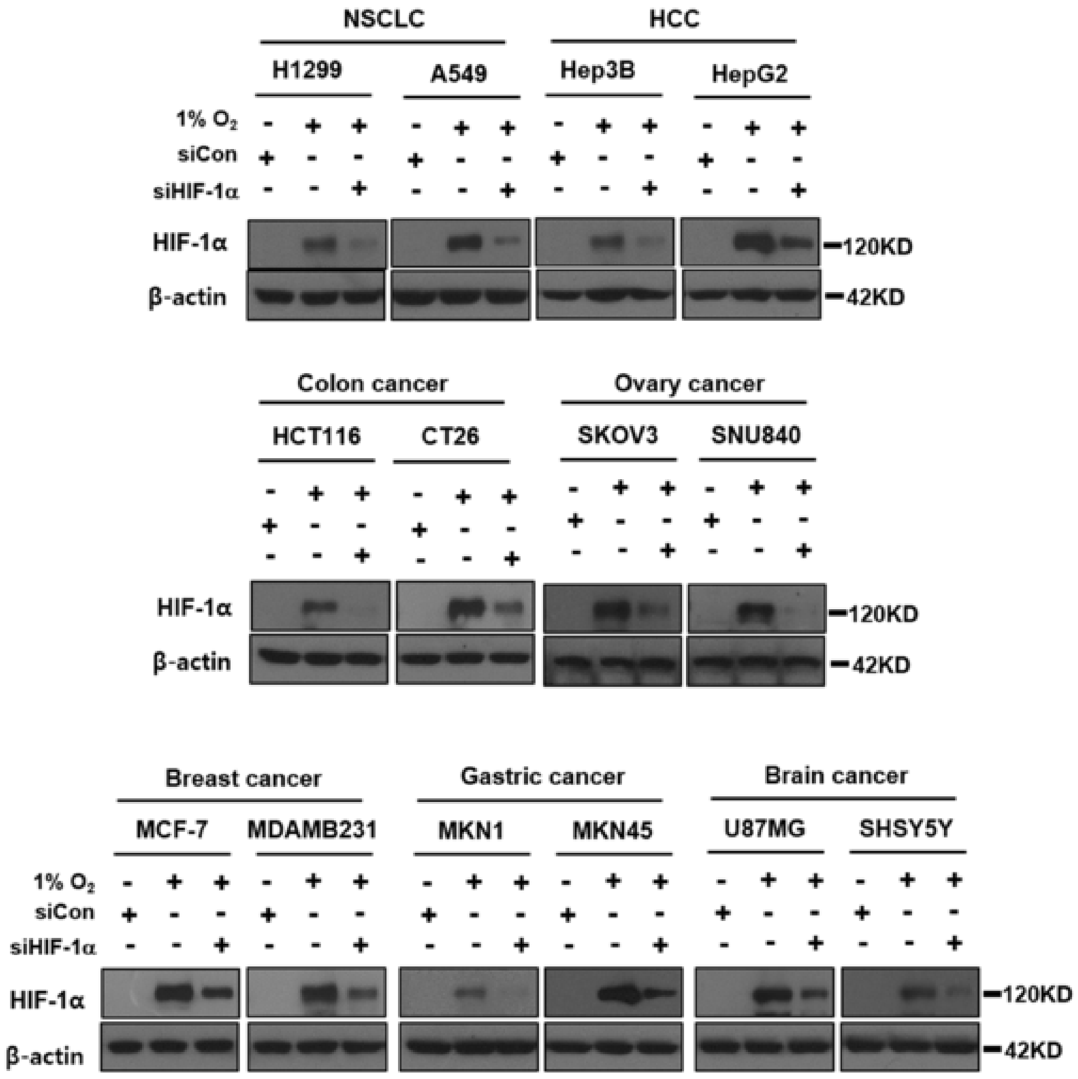

Figure S3. Expression levels of HIF-1α. H1299 and A549 (NSCLC); Hep3B, HepG2 (HCC); HCT116, CT26 (Colon cancer); SKOV3, SNU840 (Ovary cancer); MCF-7, MDAMB231 (Breast cancer); MKN1, MKN45 (Gastric cancer); and U87MG, SHSY5Y (Brain cancer) cells were transfected with siCon and siHIF-1α under normoxic or hypoxic conditions. HIF-1α and β-actin expressions were analyzed by immunoblotting.

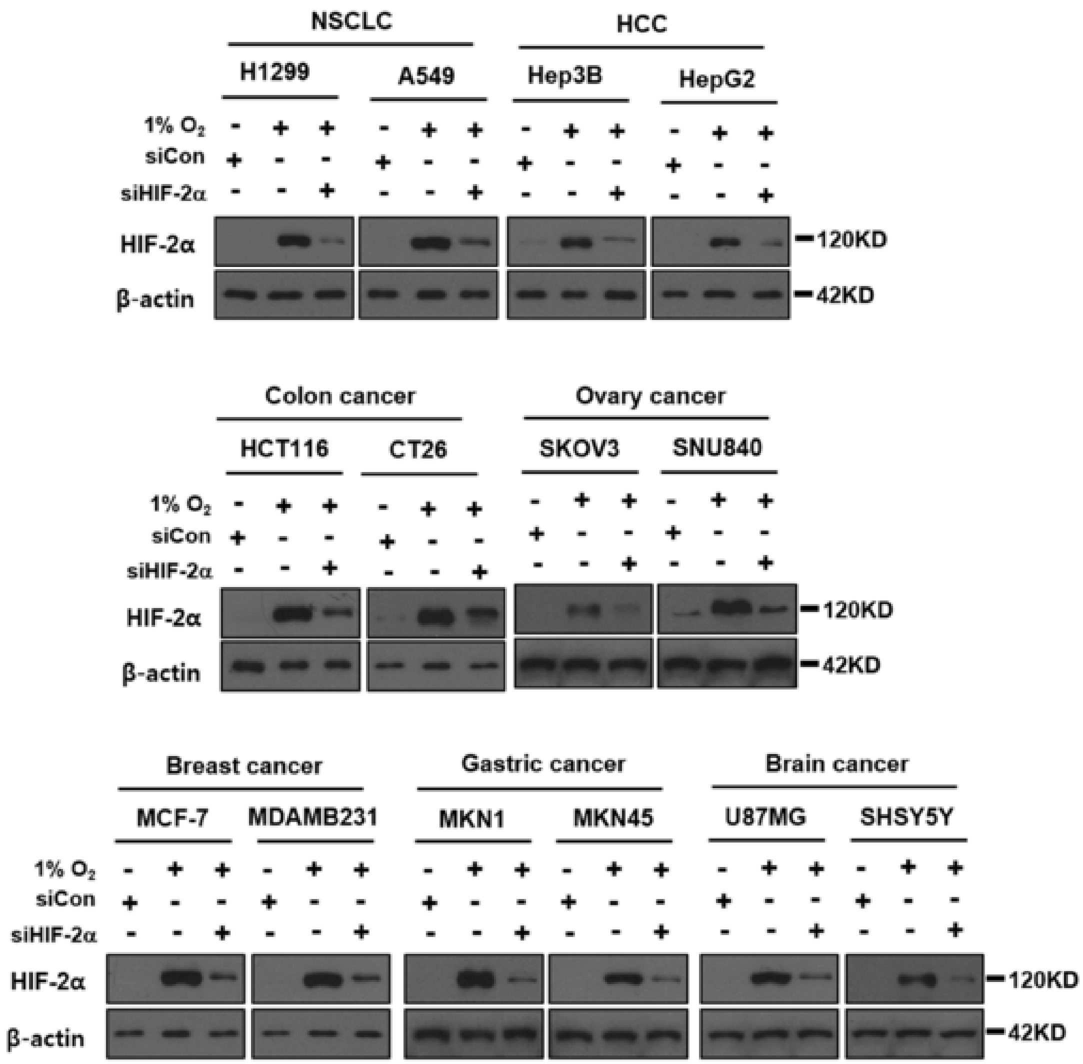

Figure S4. Expression of HIF-2α. The same cells as mentioned in Figure S3 were transfected with siCon and siHIF-2α under normoxic or hypoxic conditions. HIF-2α and β-actin expressions were analyzed by immunoblotting.

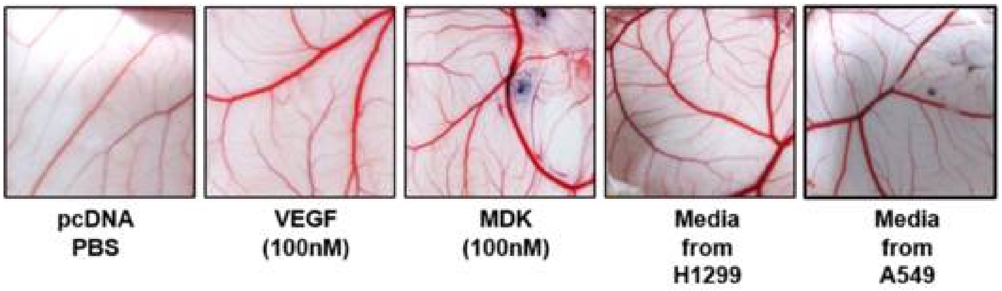

Figure S5. Images displaying angiogenic activity of MDK in CAM. CM were collected from H1299 and A549 overexpressing MDK. To measure angiogenic activity of MDK, PBS, rhVEGF165 (100ng/ml, positive control), rhMDK (100ng/ml) and CM from H1299 and A549 were added on Day 0. The images are presented as representative blood vessel branches for each group of CAM at Day 5 of incubation.

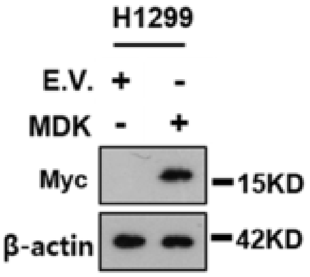

Figure S6. H1299 cells were transfected with E.V. and Myc-DDK-MDK which were selected by G418 (400 µg/ml) until colony formation. Expressing E.V. and MDK were analyzed by immunoblotting.

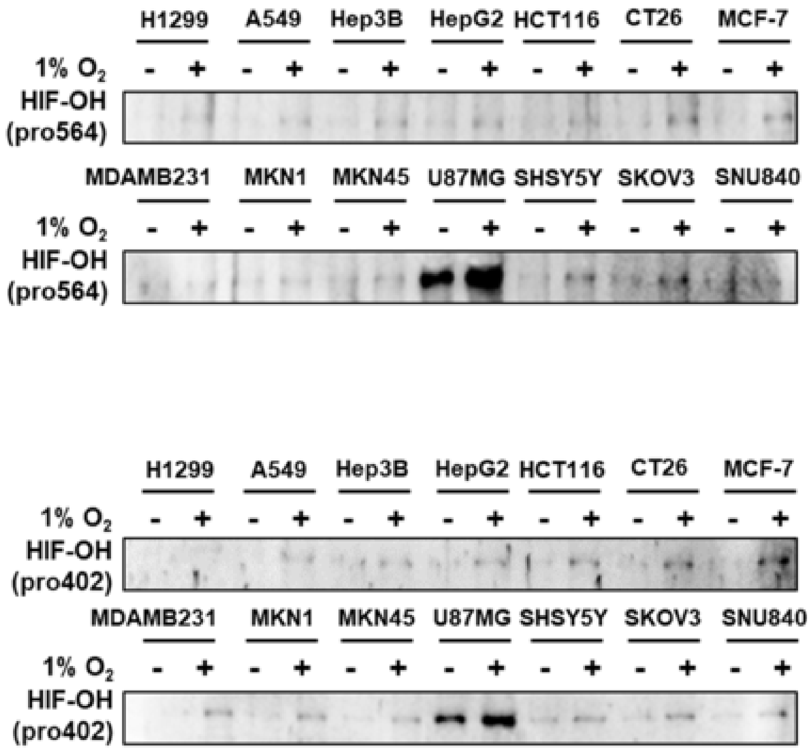

Figure S7. Expression levels Pro564 and Pro402 of HIF-1α. H1299, A549 (NSCLC); Hep3B, HepG2 (HCC); HCT116, CT26 (Colon cancer); MCF7, MDAMB231 (Breast cancer); MKN1, MKN45 (Gastric cancer); U87MG, SHSY5Y (Brain cancer); and SKOV3, SNU840 (Ovary cancer) cells were incubated for 16 h under normoxic (20% O_2) or hypoxic (1% O_2) conditions. Pro564 and Pro402 of HIF-1α and β-actin expressions were analyzed by immunoblotting.

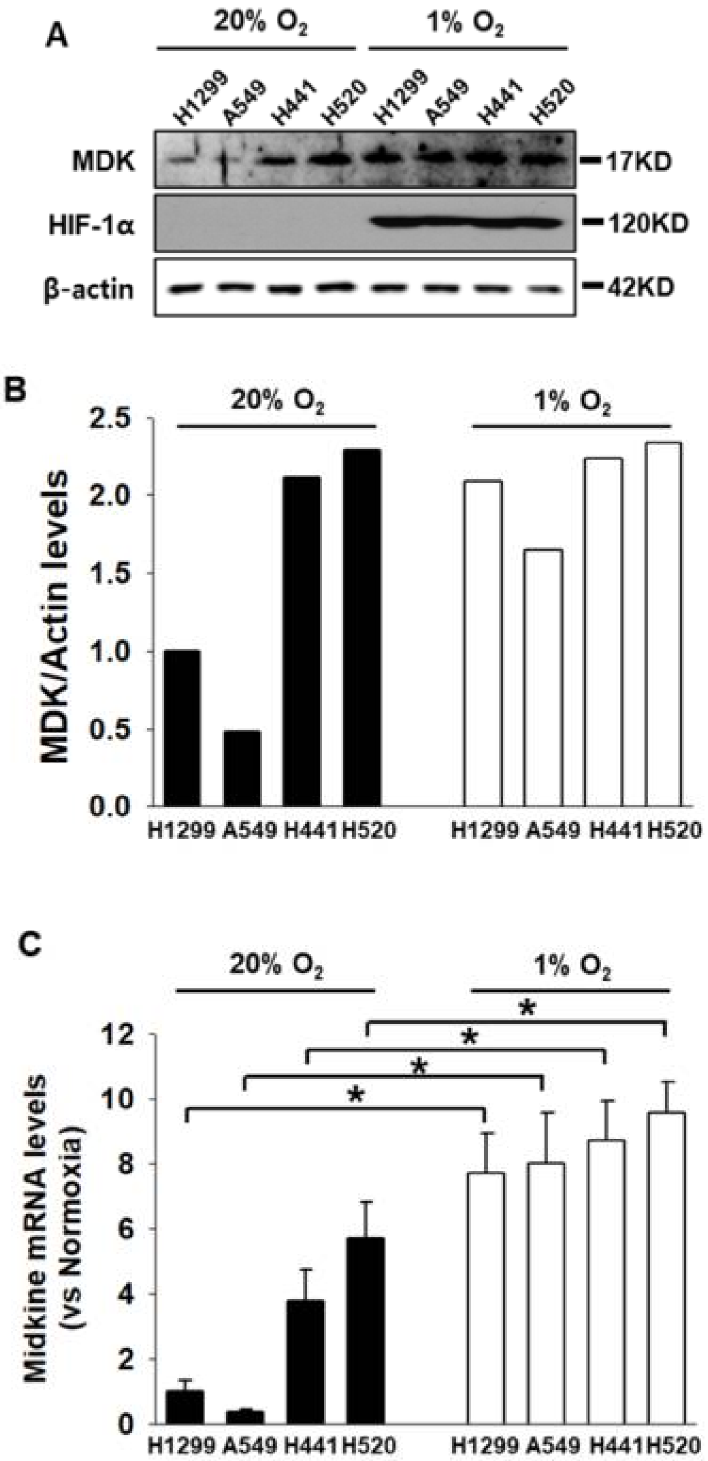

Figure S8. Measurements of protein and mRNA levels of MDK under normoxic (20% O_2) or hypoxic (1% O_2) conditions with H1299, A549, H441, and H520. A. Immunoblotting of MDK, HIF-1α, and β-actin. B. Graph of MDK levels normalized by β-actin levels. C. mRNA levels of MDK. Student's *t* test was performed and values displayed as average ±SD; $n = 6$, * $p < 0.05$.

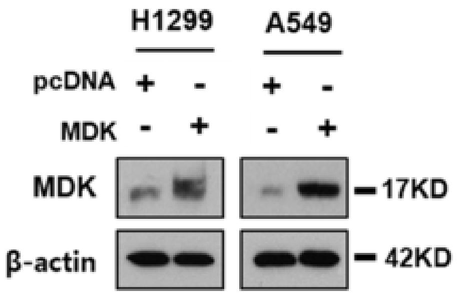

Figure S9. Expression levels of MDK. H1299 and A549 cells were transfected with pcDNA and MDK and then MDK and β-actin expressions were analyzed by immunoblotting.

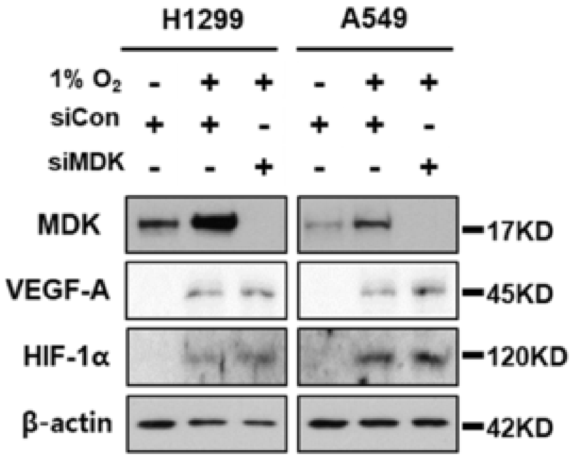

Figure S10. MDK did not regulate VEGF-A. H1299 and A549 cells were transfected with siCon and siMDK under normoxic or hypoxic conditions. MDK, VEGF-A, HIF-1α, and β-actin expressions were analyzed by immunoblotting.

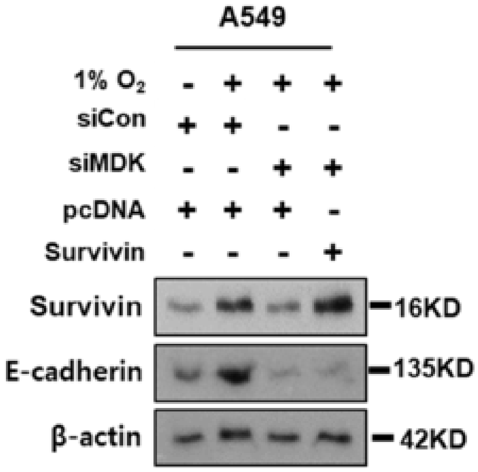

Figure S11. Survivin did not regulate EMT signaling. A549 cells were transfected with siCon, siMDK, pcDNA, and survivin. Survivin, E-cadherin, and β-actin expressions were analyzed by immunoblotting.

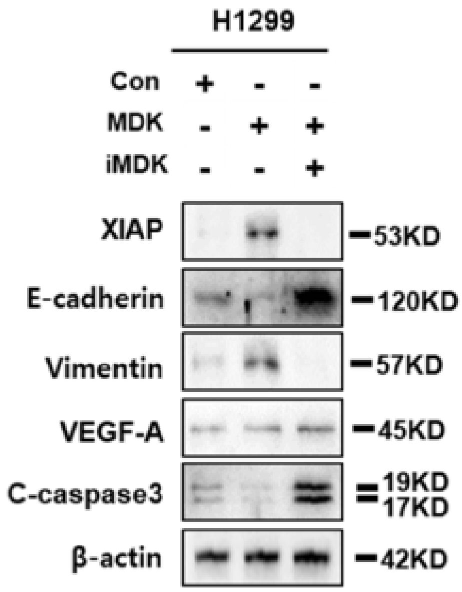

Figure S12. Western blotting with xenograft in vivo samples. Con, MDK, and MDK+iMDK samples from H1299 xenograft in vivo model in Figure 5. analyzed XIAP, E-cadherin, vimentin, VEGF-A, cleaved-caspase 3 and β-actin by using Immunoblotting assay.

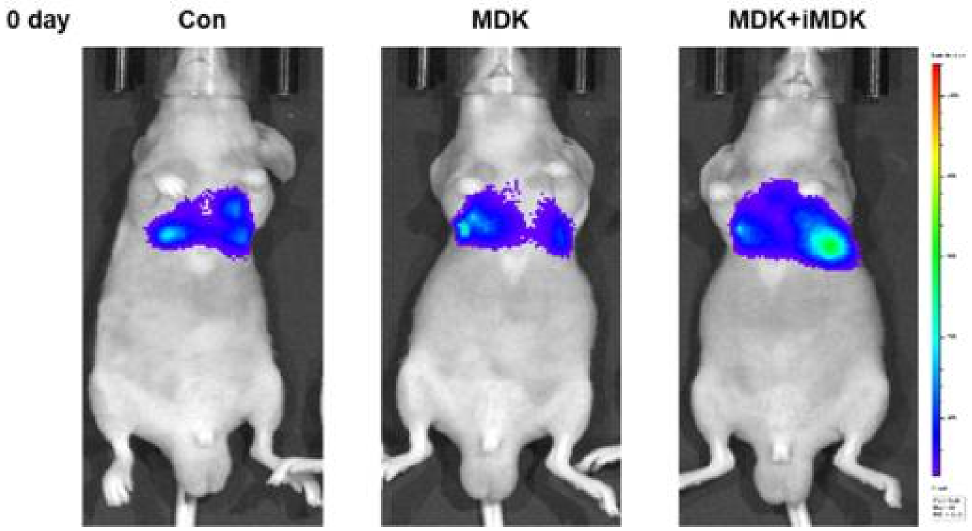

Figure S13. Representative Bio-luminescence images at 0 days for control, MDK, and iMDK are shown, revealing a BLI signal originating from the site of injection.

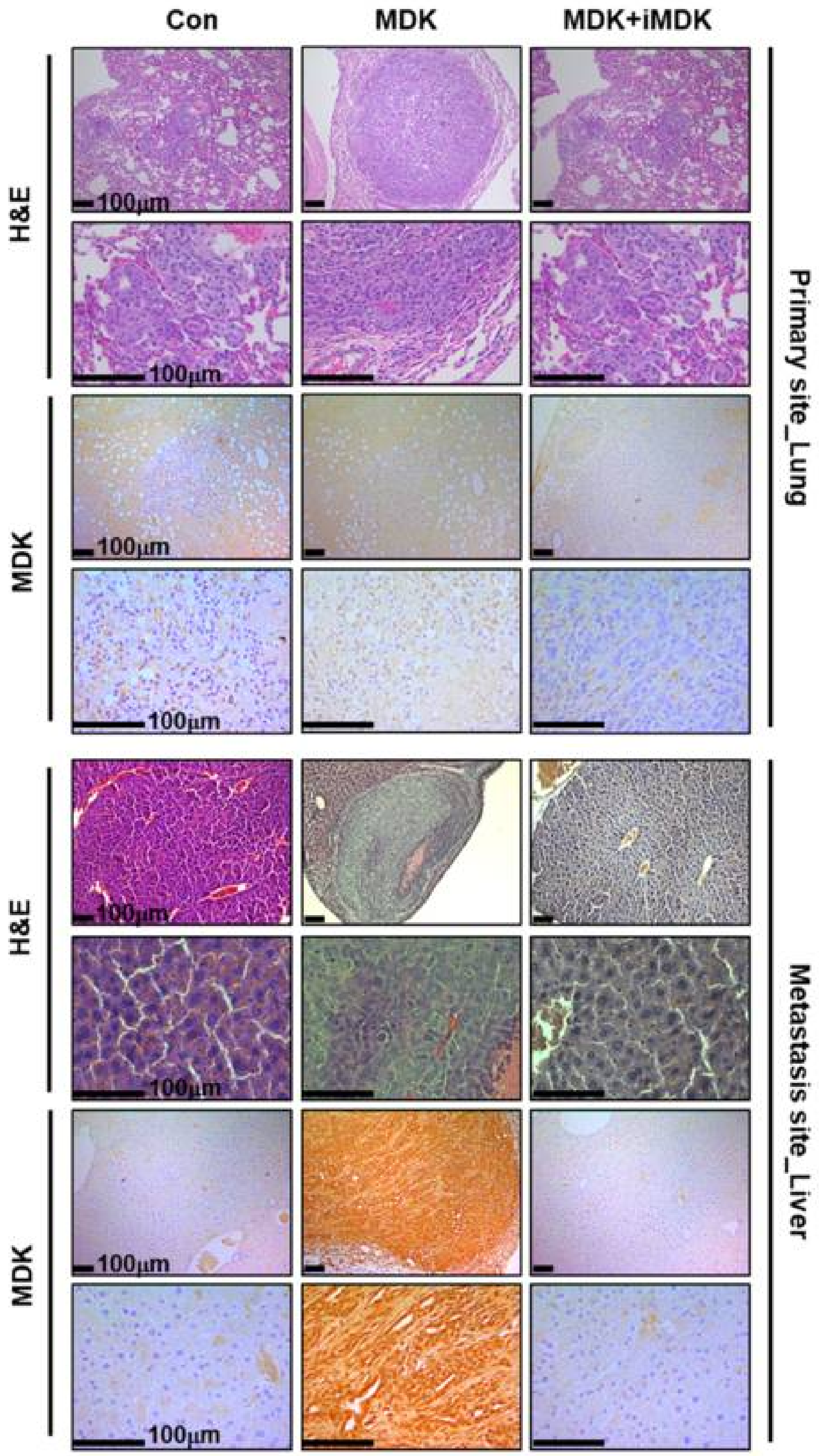

Figure S14. Immunohistochemical staining with H&E and MDK with primary site (lung) and metastasis site (liver) in Figure 7.

Catalog No.: NCC0720-1

Institute	Researcher Name	Cell Line ID	Date Received	Date Analyzed
National Cancer Center	Dong Hoon Shin	NCI-H1299	07-17-20	07-24-20

Morphology: Epithelial

Culture properties: Adherent

Culture Method: RPMI-1640 10% FBS

Subcultivation Ration: 1:3~1:6; Twice per week

Trypsined Time: 3-5 minutes

Results:

Database Match Result(s)				
	Designation	Cell Number	% Match	Database
1	NCI-H1299	$1x10^6$ cells	100	DSMZ

STR profiles:

STR Profile	AMEL	CSF1PO	D13S317	D16S539	D55818	D7S820	TH01	TPOX	vWA
NCI-H1299	X	12	12	12 13	11	10	69.3	8	16 17 18

Catalog No.: NCC0720-2

Institute	Researcher Name	Cell Line ID	Date Received	Date Analyzed
National Cancer Center	Dong Hoon Shin	A549	07-17-20	07-24-20

Morphology: Epithelial

Culture properties: Adherent

Culture Method: F12-K/RPMI-1640 10% FBS

Subcultivation Ration: 1:3~1:8; Twice per week

Trypsined Time: 3-5 minutes

Results:

Database Match Result(s)				
	Designation	Cell Number	% Match	Database
1	A549	$1x10^6$ cells	100	DSMZ

STR profiles:

STR Profile	AMEL	CSF1PO	D13S317	D16S539	D55818	D7S820	TH01	TPOX	vWA
NCI-H1299	X	9 10	8	11 12	11 12	9 11	9.3	8 9	16

Figure S15. The authentication of the H1299 and A549 cell line. PCR implemented with STR multi-amplification KIT (PowerPlexTM 16HS System). No loci had tri-alleles or tetra-alleles. Contamination of other human cells was not detected. 100% matched cell line H1299 and A549 were found in both ATCC and DSMZ data banks.

© 2020 by the authors. Licensee MDPI, Basel, Switzerland. This article is an open access article distributed under the terms and conditions of the Creative Commons Attribution (CC BY) license (http://creativecommons.org/licenses/by/4.0/).
