# Supplementary material for: Midkine Is a Potential Therapeutic Target of Tumorigenesis, Angiogenesis, and Metastasis in Non-Small Cell Lung Cancer
Source: Cancers (Basel). 2020 Aug 24;12(9):2402. doi: 10.3390/cancers12092402 (PMC7563676; doi:10.3390/cancers12092402)
Supplement: Supplementary file 1 [file cancers-12-02402-s001.zip › Figure S16.pdf]

Figure 2.

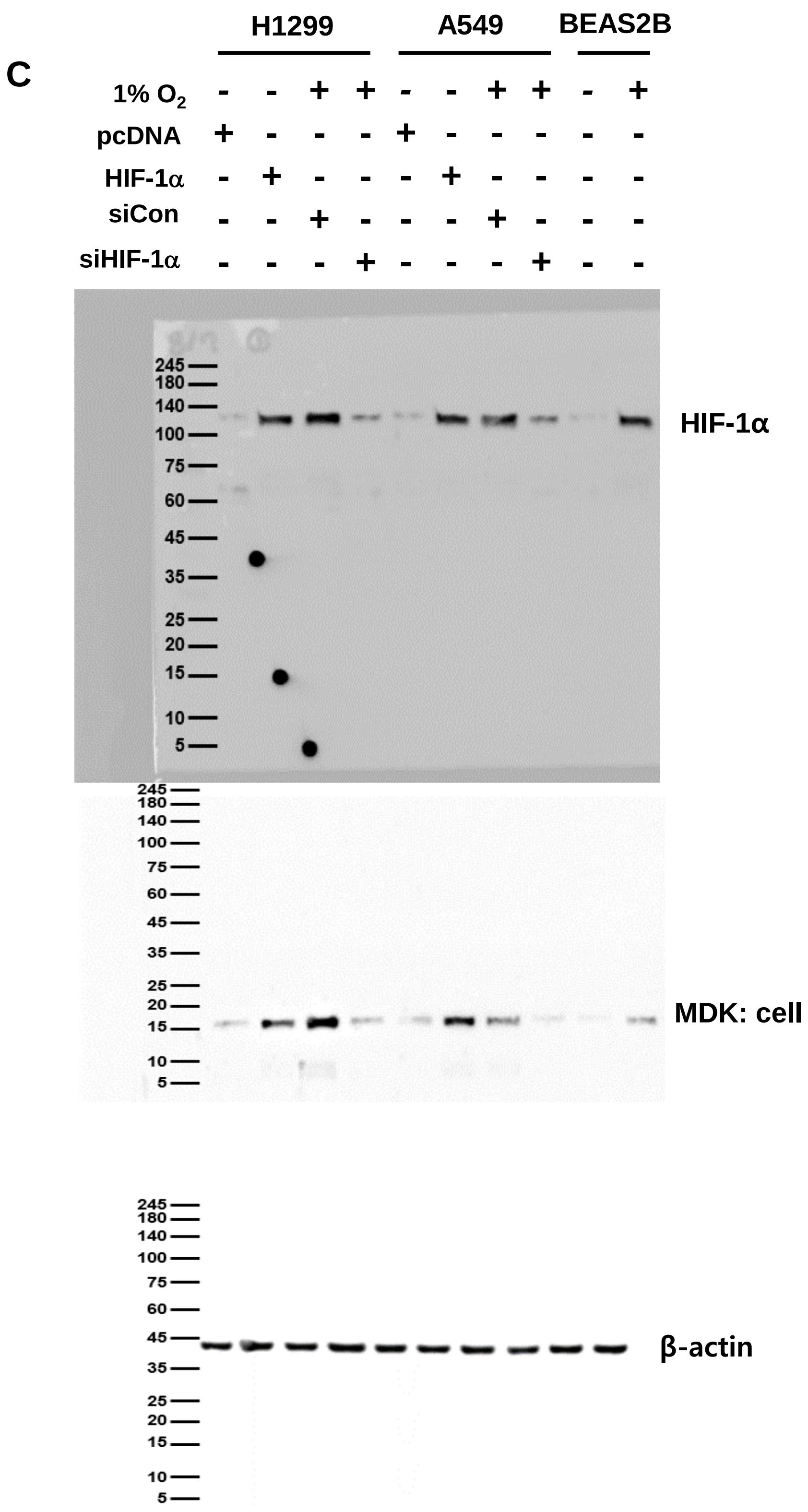
C
H1299
A549
BEAS2B
1% O2
pcDNA
HIF-1α
siCon
siHIF-1α
HIF-1α
MDK: cell
β-actin

Figure 2.

C

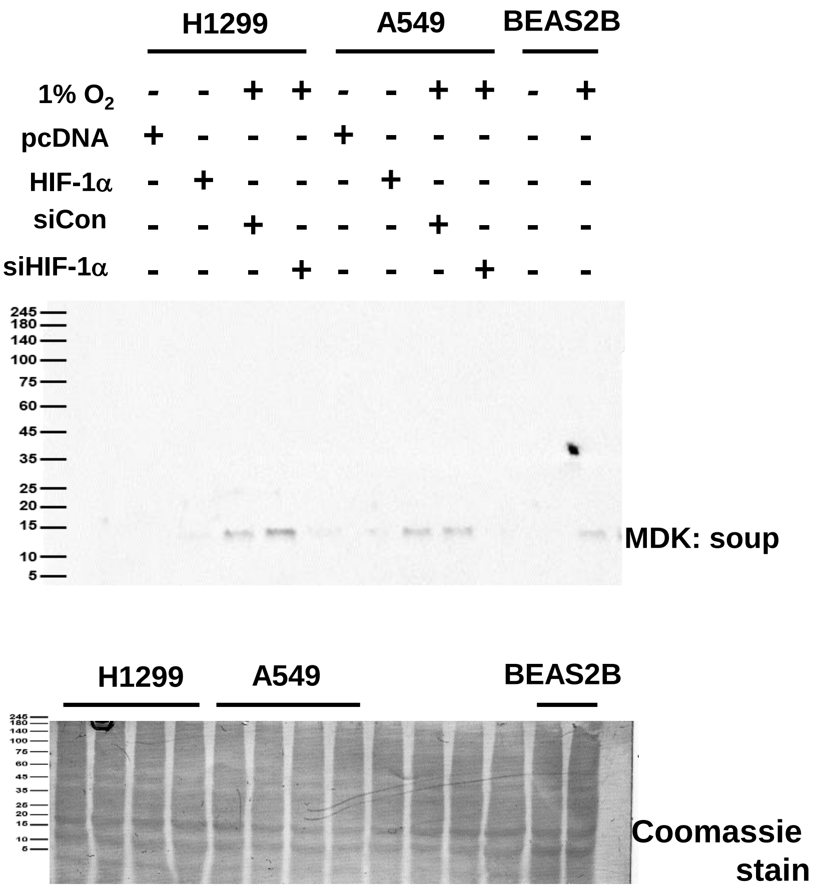
H1299
A549
BEAS2B
1% O2
pcDNA
HIF-1α
siCon
siHIF-1α
245
180
140
100
75
60
45
35
25
20
15
10
5
MDK: soup
H1299
A549
BEAS2B
Coomassie stain

Figure 4.

**B**

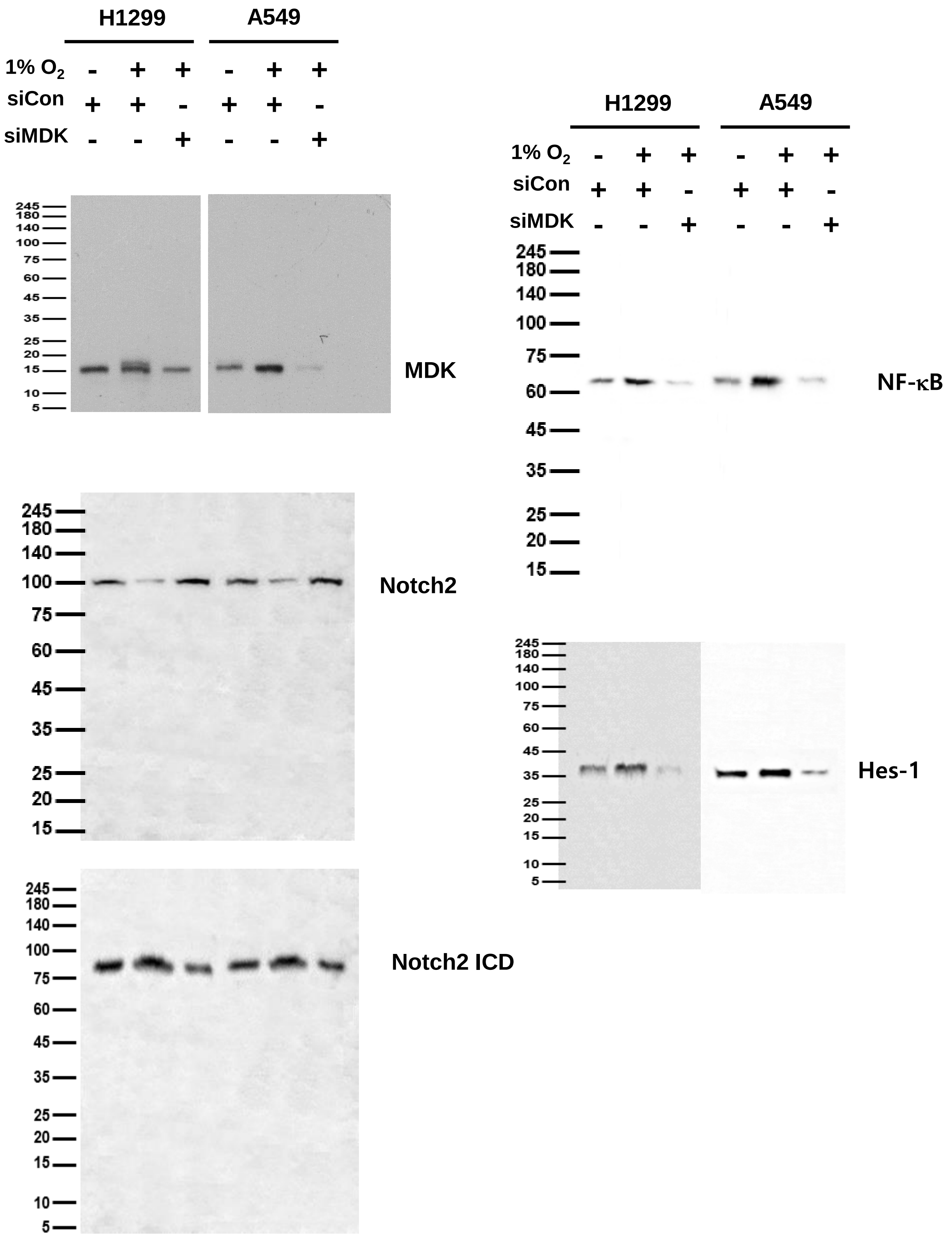
H1299
A549
1% $O_2$ - + + - + +
siCon + + - + + -
siMDK - - + - - +
245
180
140
100
75
60
45
35
25
20
15
10
5
MDK
Notch2
Notch2 ICD
NF-κB
Hes-1

Figure 4.

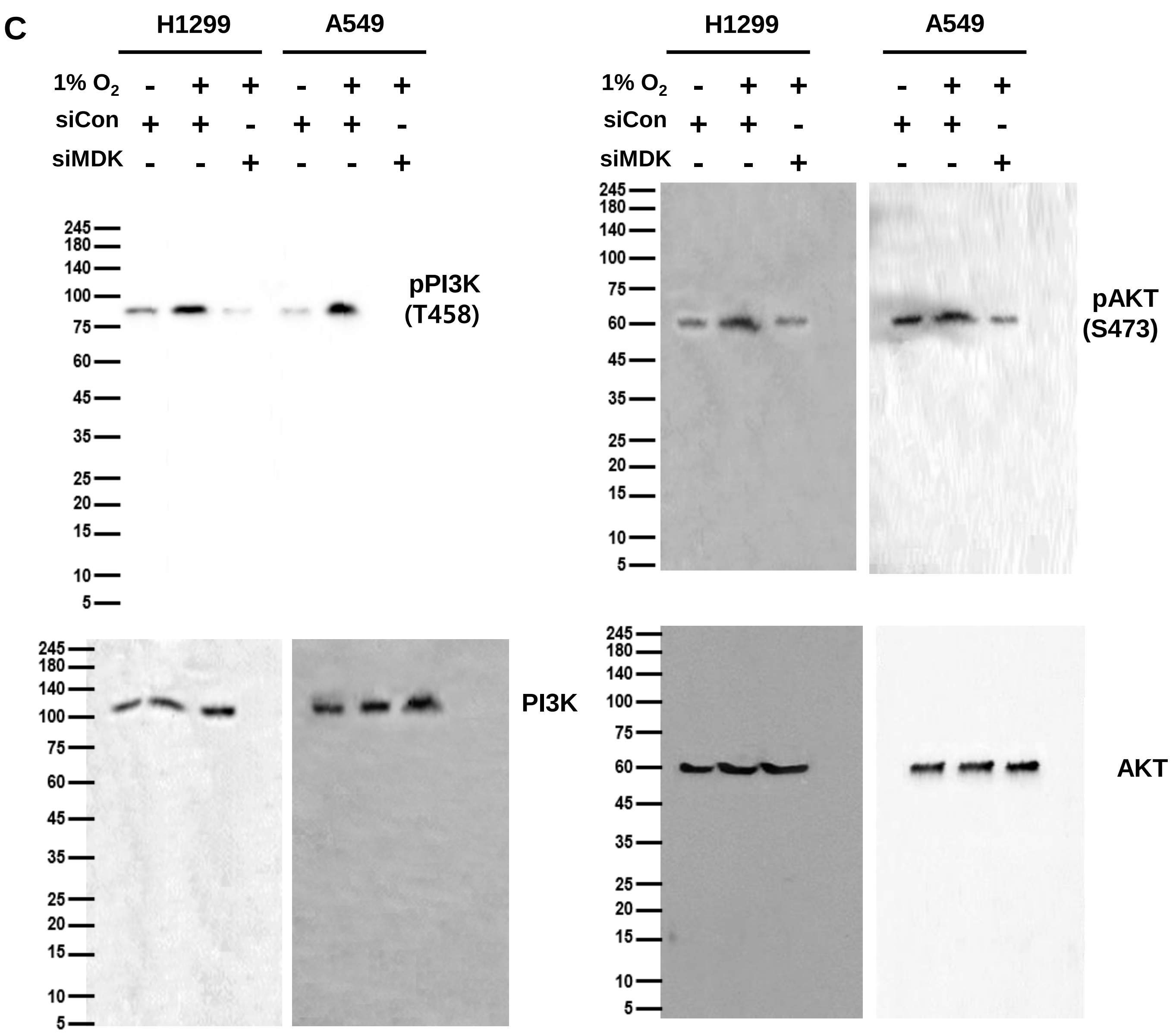
C
H1299
A549
1% $O_2$
siCon
siMDK
pPI3K (T458)
PI3K
pAKT (S473)
AKT
245
180
140
100
75
60
45
35
25
20
15
10
5

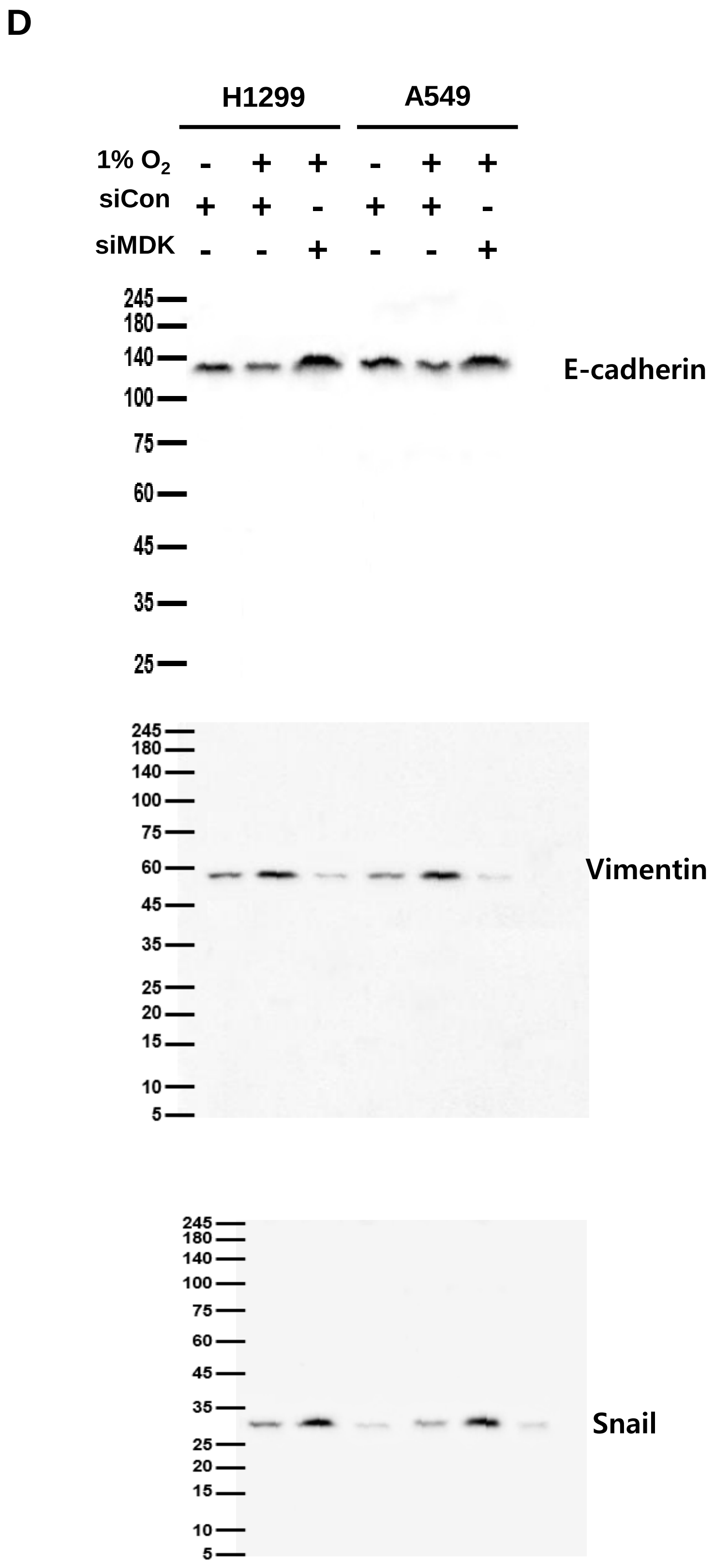
D
H1299
A549
1% $O_2$ - + + - + +
siCon + + - + + -
siMDK - - + - - +
245
180
140
100
75
60
45
35
25
E-cadherin
245
180
140
100
75
60
45
35
25
20
15
10
5
Vimentin
245
180
140
100
75
60
45
35
25
20
15
10
5
Snail

Figure 4.

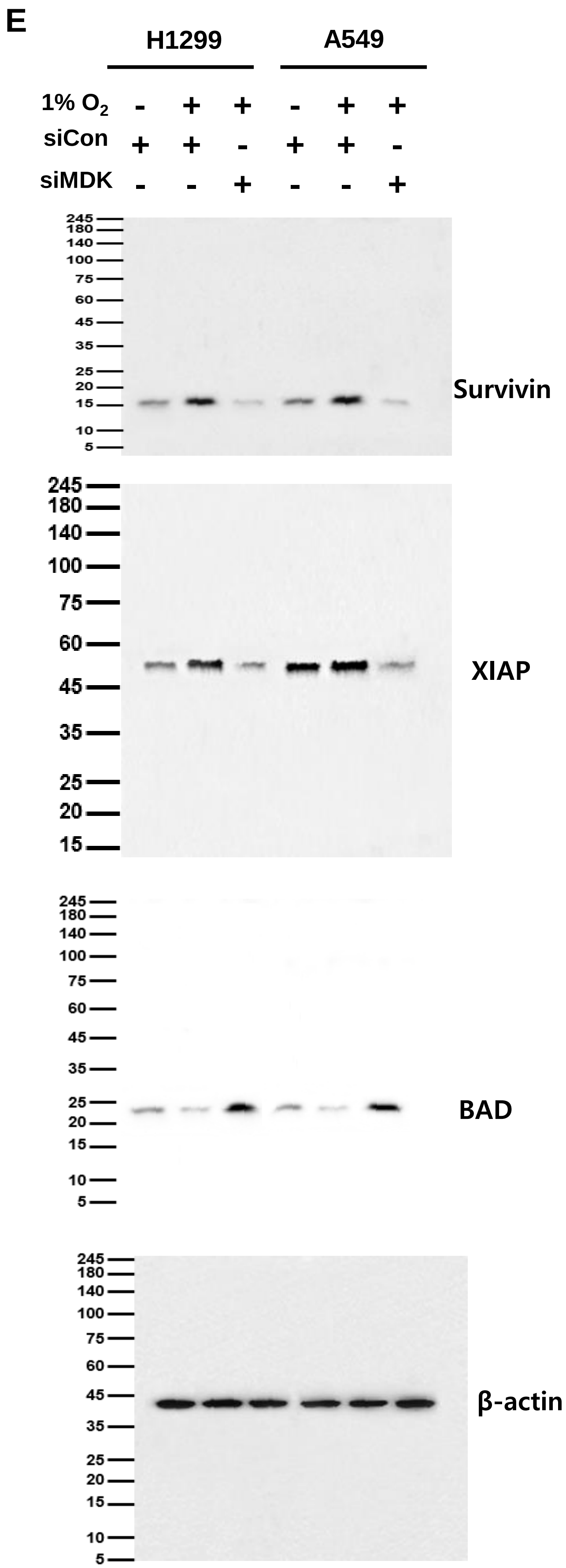
E
H1299
A549
1% O2 - + + - + +
siCon + + - + + -
siMDK - - + - - +
Survivin
XIAP
BAD
β-actin
245
180
140
100
75
60
45
35
25
20
15
10
5

**Figure S1.**

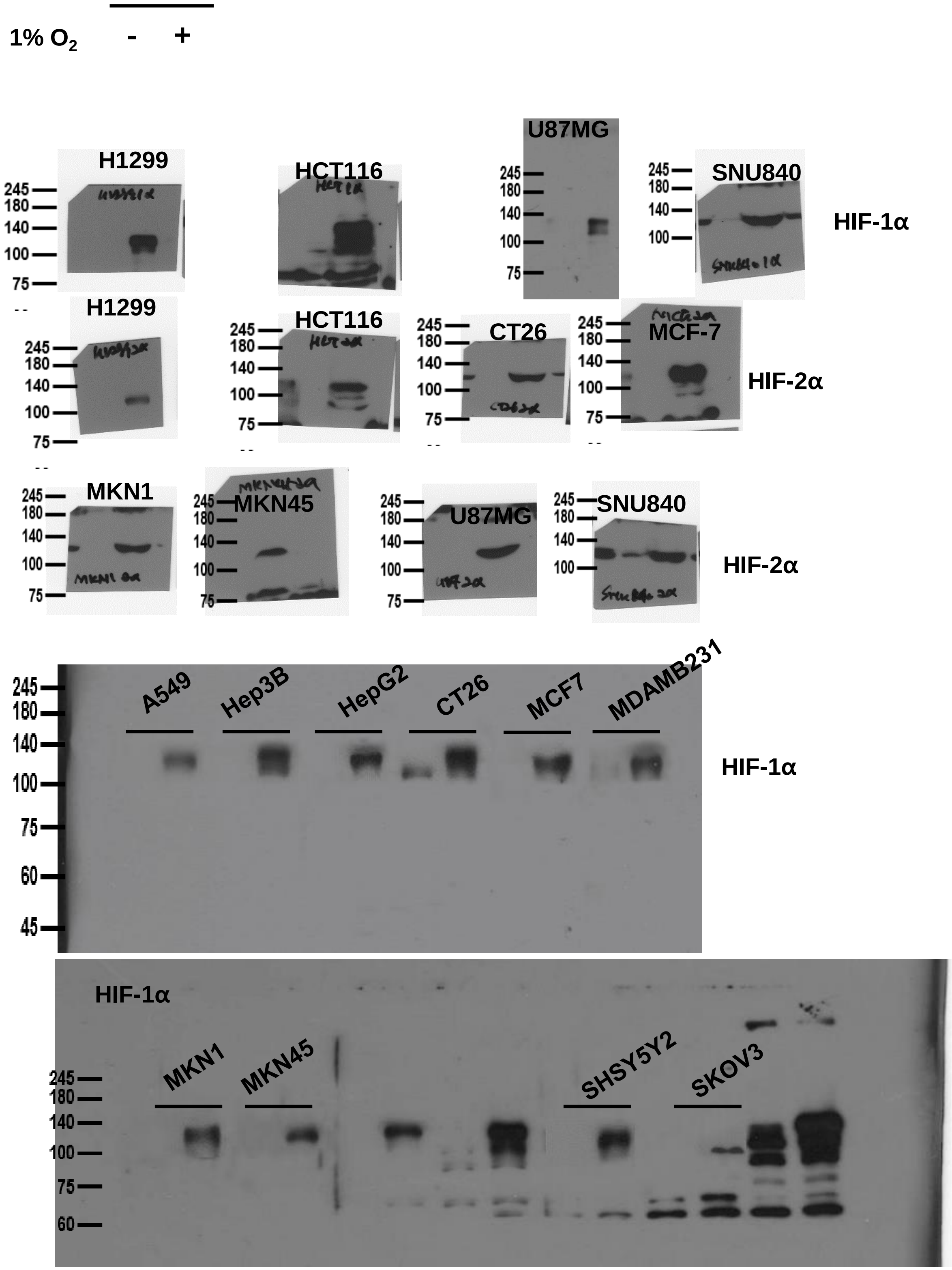
1% $O_2$ - +
H1299
HCT116
U87MG
SNU840
HIF-1α
H1299
HCT116
CT26
MCF-7
HIF-2α
MKN1
MKN45
U87MG
SNU840
HIF-2α
A549
Hep3B
HepG2
CT26
MCF7
MDAMB231
HIF-1α
HIF-1α
MKN1
MKN45
SHSY5Y2
SKOV3
245
180
140
100
75
60
45

**Figure S1.**

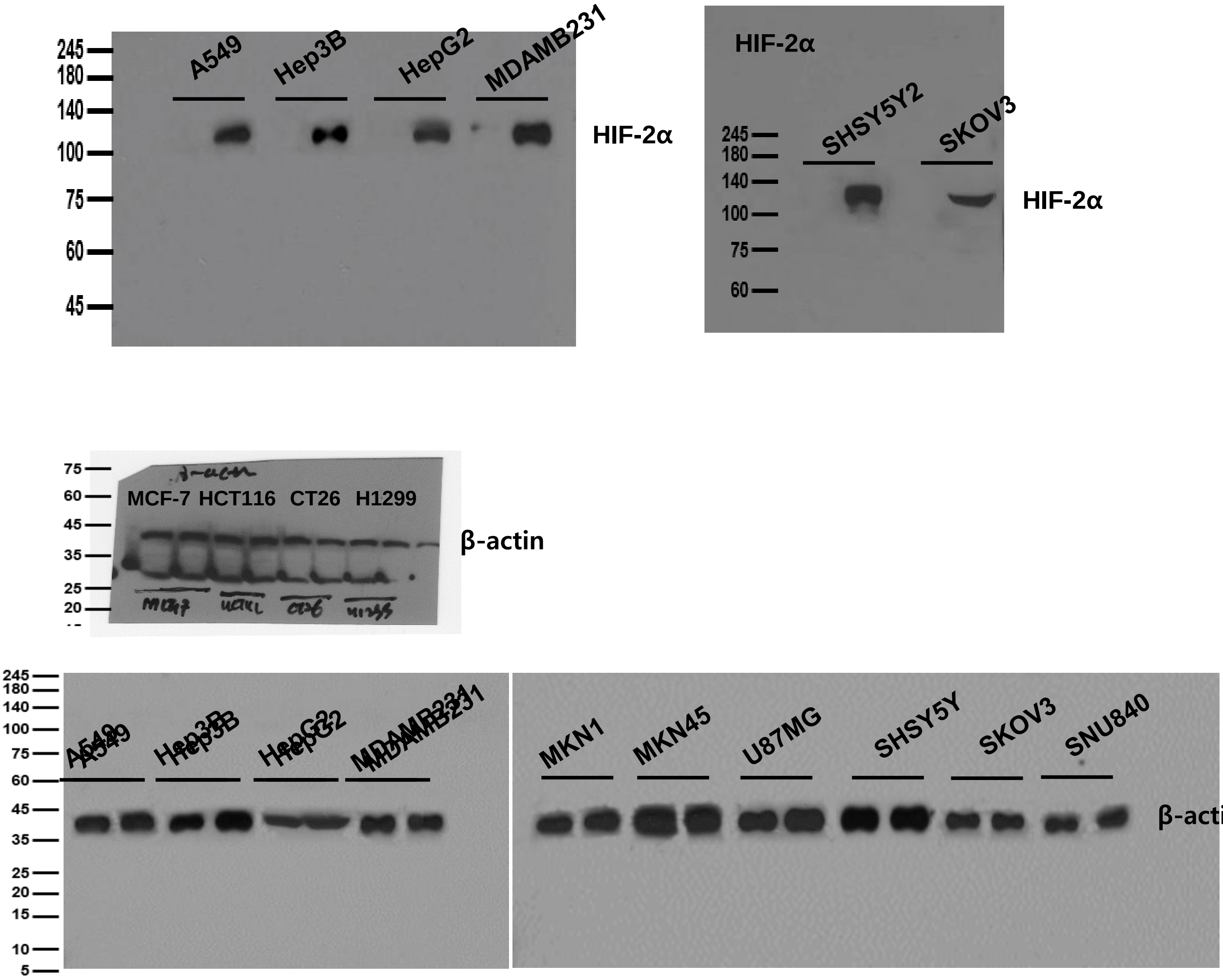

245
180
140
100
75
60
45
A549
Hep3B
HepG2
MDAMB231
HIF-2α
HIF-2α
SHSY5Y2
SKOV3
245
180
140
100
75
60
HIF-2α
75
60
45
35
25
20
MCF-7
HCT116
CT26
H1299
β-actin
245
180
140
100
75
60
45
35
25
20
15
10
5
A549
Hep3B
HepG2
MDAMB231
MKN1
MKN45
U87MG
SHSY5Y
SKOV3
SNU840
β-acti

**Figure S3.**

| | | | |
|---|---|---|---|
| 1% $O_2$ | - | + | + |
| siCon | + | - | - |
| siHIF-1α | - | - | + |

HIF-1α

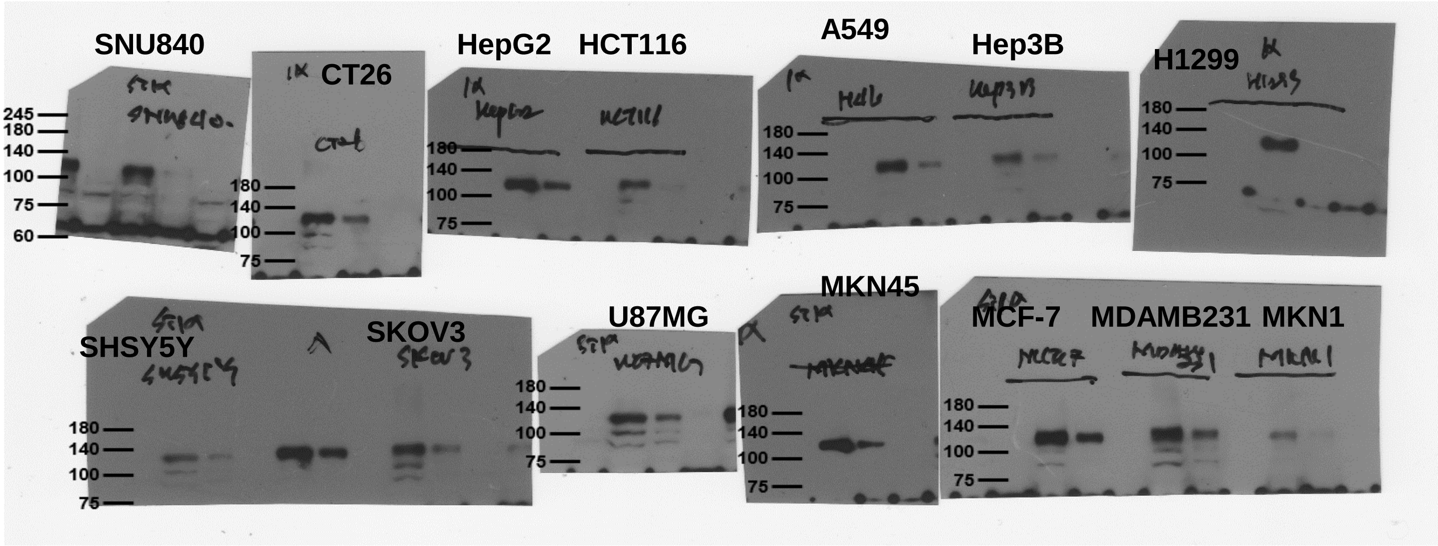


β-actin

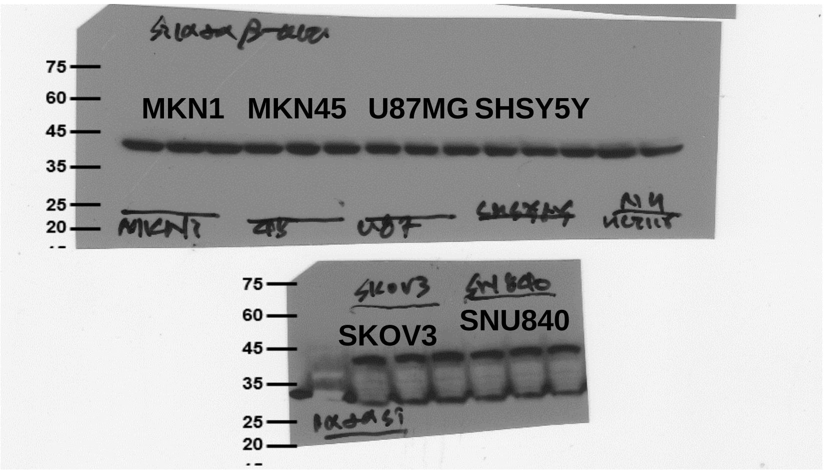


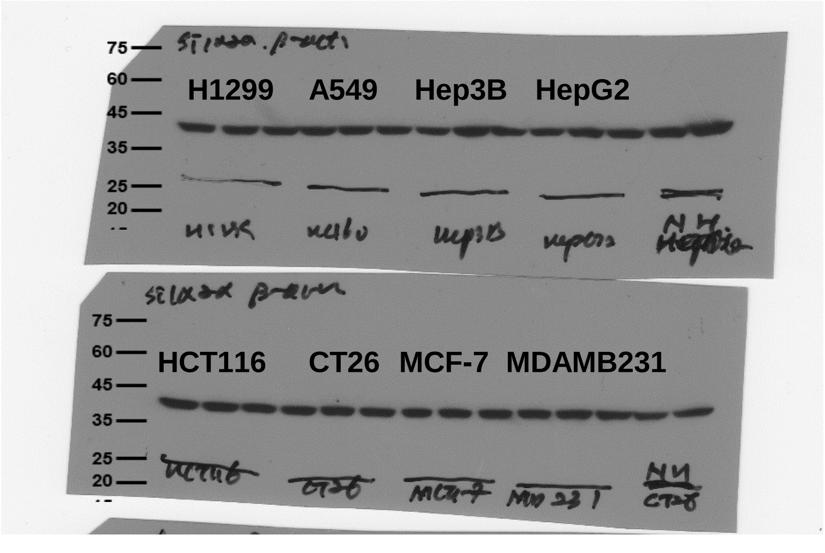

**Figure S4.**

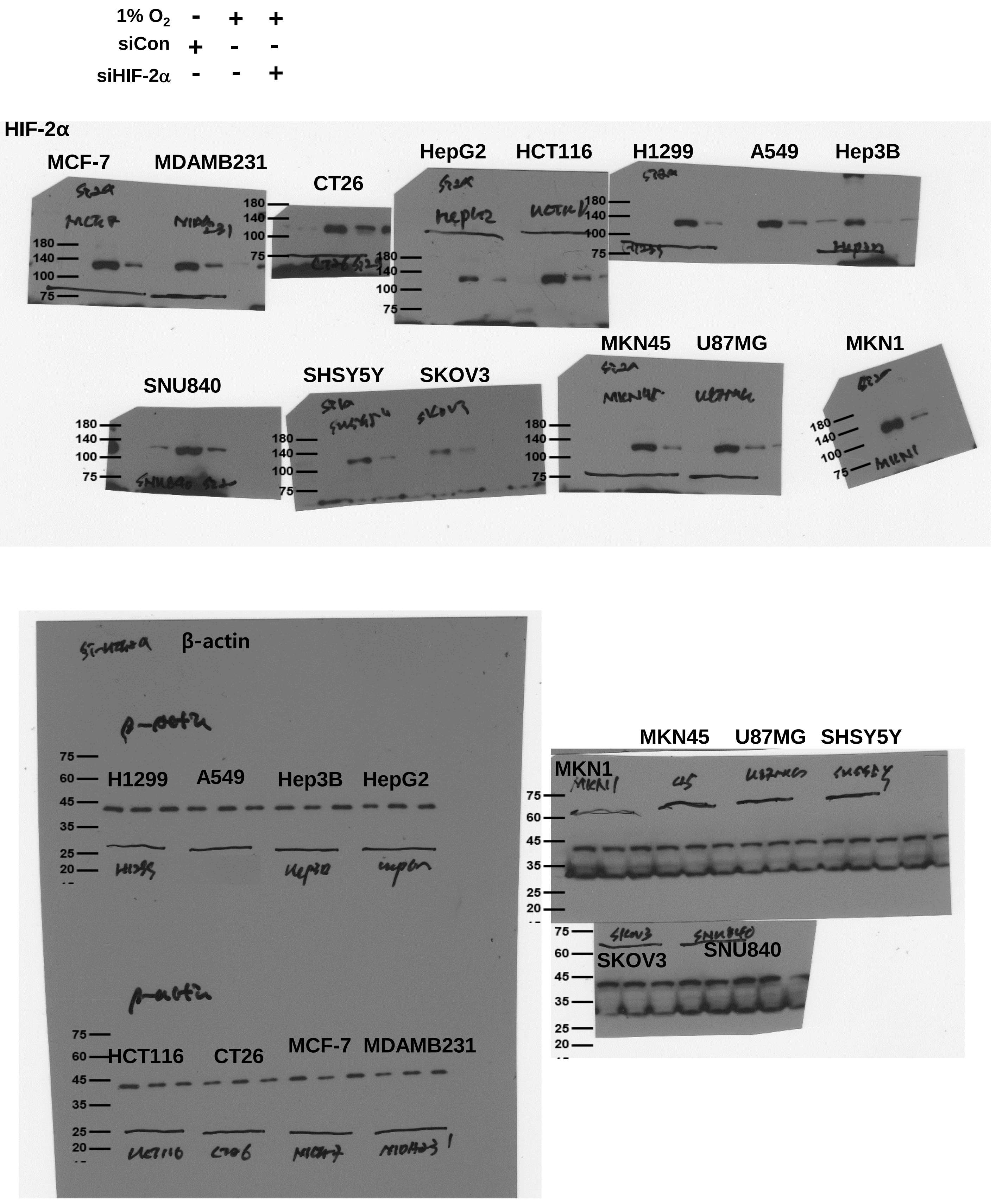

1% O2 - + +
siCon + - -
siHIF-2α - - +
HIF-2α
MCF-7
MDAMB231
CT26
HepG2
HCT116
H1299
A549
Hep3B
SNU840
SHSY5Y
SKOV3
MKN45
U87MG
MKN1
β-actin
H1299
A549
Hep3B
HepG2
HCT116
CT26
MCF-7
MDAMB231
MKN45
U87MG
SHSY5Y
MKN1
SKOV3
SNU840

**Figure S6.**

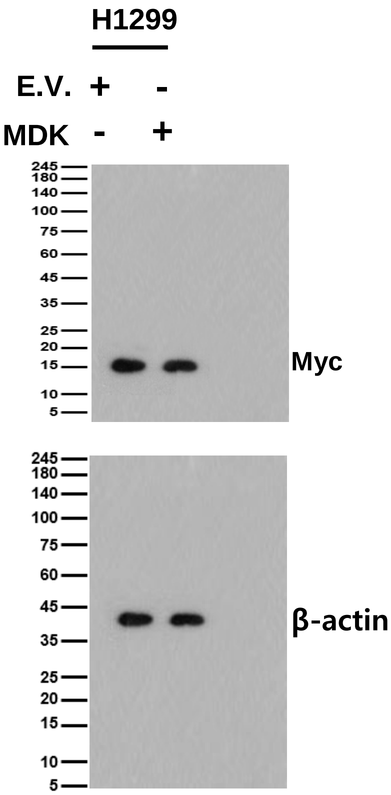
H1299
E.V. + -
MDK - +
245
180
140
100
75
60
45
35
25
20
15
10
5
Myc
245
180
140
100
75
60
45
35
25
20
15
10
5
β-actin
